# Novel Flavivirus Attenuation Markers Identified in the Envelope Protein of Alfuy Virus

**DOI:** 10.3390/v13020147

**Published:** 2021-01-20

**Authors:** Daniel Westlake, Helle Bielefeldt-Ohmann, Natalie A. Prow, Roy A. Hall

**Affiliations:** 1 School of Chemistry and Molecular Biosciences, The University of Queensland, St. Lucia, QLD 4072, Australia; daniel.westlake@uqconnect.edu.au (D.W.); h.bielefeldtohmann1@uq.edu.au (H.B.-O.); 2 Australian Infectious Diseases Research Centre, The University of Queensland, St. Lucia, QLD 4072, Australia; 3 School of Veterinary Sciences, The University of Queensland, St. Lucia, QLD 4072, Australia; 4 Experimental Therapeutics Laboratory, School of Clinical and Health Sciences, University of South Australia Cancer Research Institute, Adelaide, SA 5000, Australia

**Keywords:** Alfuy virus (ALFV), glycosaminoglycan (GAG), flavivirus, attenuation, Murray Valley encephalitis virus (MVEV)

## Abstract

Alfuy (ALFV) is an attenuated flavivirus related to the Murray Valley encephalitis virus (MVEV). We previously identified markers of attenuation in the envelope (E) protein of the prototype strain (ALFV_3929_), including the hinge region (E273–277) and lack of glycosylation at E154-156. To further determine the mechanisms of attenuation we assessed ALFV_3929_ binding to glycosaminoglycans (GAG), a known mechanism of flaviviruses attenuation. Indeed, ALFV_3929_ exhibited reduced binding to GAG-rich cells in the presence of heparin; however, low-passage ALFV isolates were relatively unaffected. Sequence comparisons between ALFV strains and structural modelling incriminated a positively-charged residue (K327) in ALFV_3929_ as a GAG-binding motif. Substitution of this residue to the corresponding uncharged residue in MVEV (L), using a previously described chimeric virus containing the prM & E genes of ALFV_3929_ in the backbone of MVEV (MVEV/ALFV-prME), confirmed a role for K327 in enhanced GAG binding. When the wild type residues at E327, E273–277 and E154–156 of ALFV_3929_ were replaced with the corresponding residues from virulent MVEV, it revealed each motif contributed to attenuation of ALFV_3929_, with the E327/E273–277 combination most dominant. These data demonstrate that attenuation of ALFV_3929_ is multifactorial and provide new insights for the rational design of attenuated flavivirus vaccines.

## 1. Introduction

Alfuy virus (ALFV) is a member of the Japanese encephalitis virus (JEV) sero-complex of flaviviruses and is closely related genetically and antigenically to the pathogenic Murray Valley encephalitis virus (MVEV), which is associated with encephalitis in humans and equines [1]. However, while it shares 84% deduced amino acid sequence identity with MVEV (89% in the viral envelope (E) protein), ALFV is neither pathogenic in humans nor neuro-invasive in mice [1,2].

ALFV is a positive sense RNA virus with a genome of 11.4kb, encoding a single polyprotein co- and post-translationally processed into three structural proteins, capsid (C), pre-membrane/membrane (prM/M) and envelope (E), in addition to seven non-structural proteins, NS1, NS2A, NS2B, NS3, NS4A, NS4B and NS5. The genome also includes untranslated regions at the 3′ and 5′ ends. The mature virion is comprised of an icosohedral protein structure consisting of prM/M and E embedded in a host acquired lipid envelope. Inside the outer layer is the nucleocapsid consisting of the C protein and viral RNA [3]. The E protein is a class II fusion protein involved in virus entry into cells by receptor-mediator endocytosis [4,5,6] and low-pH-dependent fusion of the virus envelope with endosomal membranes [7,8,9,10,11]. The flavivirus E protein can be divided into three domains (I, II and III), with virulence determinants identified within each of these, including the N-linked glycosylation motif in domain I [12], the fusion peptide in domain II [13] and the RGD motif in domain III [14]. The RGD motif is a site in the MVEV envelope protein (E390) which has been shown by Lee and Lobigs to be associated GAG binding. In wild type virus this is a positively charged arginine residue; however, it is directly adjacent to a negatively charged aspartic acid residue (E392), negating the net positive charge and ablating GAG binding [14].

In our previous study we identified residues in the envelope (E) protein in ALFV at the conserved glycosylation site (E154–156) and hinge region (E273–277) as possible attenuation motifs [15]. However, the picture was still incomplete, with substitutions at these sites with corresponding residues from its virulent MVEV cousin, only partially restoring neuro-invasiveness to the ALFV construct [15]. The ALFV constructs used in that study exhibited a phenotype indicative of glycosaminoglycan (GAG) binding, specifically, the lack of replication or spread in the periphery in vivo, reduced haemagglutination range, a characteristic small plaque phenotype and distinctive growth kinetics in the SW13 cell line [1,15].

GAG binding was previously overlooked as a potential attenuation factor in the ALFV construct, as sequencing of the E protein had revealed no changes to the expected GAG binding motif at residue 390, which is a site associated with MVEV GAG binding [1].

There are many causes of in vitro attenuation of flaviviruses; however, one of the most universal is the enhanced affinity of the virus to GAG, a typically sulphated form of polysaccharide commonly found in the cellular membrane and in the extracellular matrix of vertebrates [16]. GAGs are long unbranched polysaccharides, consisting of a repeating disaccharide unit comprised of a hexose core hexuronic acid linked to a hexosamine [16]. GAGs are added when proteins produced in the rough endoplasmic reticulum are modified post-translationally by glycosyltransferases in the Golgi apparatus, adding GAG disaccharides to protein cores to produce proteoglycans [17,18]. Glycosaminoglycans are a key component of the connective tissues/extracellular matrix [19]. Due to their strong negative charge and the high density of the sugar molecules, GAGs are hydrophilic. The most prevalent GAG in animals is chondroitin sulphate, usually found in cartilage where it provides much of its resistance to compression [20]. Other GAGs include dermatan sulphate, found in the skin, blood vessels, heart valves, tendons and lungs [21]; keratin sulphate, found in the cornea, cartilage and bone [22]; and heparan sulphate, found in all animal tissues [23].

It has been established previously that heparan sulphate GAGs found on the cell surface can act as a receptor for various viruses including α-herpesviruses [24], HIV-1 [25], human cytomegalovirus [26], foot and mouth disease virus [27], vaccinia virus [28], and adeno-associated virus type 2 [29]. GAG has also been shown to act as a virion concentration attachment molecule on the cell surface [30]. This model is supported by results involving experiments using the flaviviruses, dengue virus (DENV) and tick-borne encephalitis virus (TBEV). In vitro studies with DENV have been shown to first bind to heparan sulphate on the cell surface and then to a high affinity receptor which initiates endocytosis [31,32,33].

GAG binding motifs are characterised by large positively charged amino acids expressed on the surface of viral structural proteins, i.e., the envelope protein of flaviviruses. A number of flavivirus models for GAG binding have been developed, including DENV, TBEV, JEV and MVEV. Most relevant to this investigation are studies by Lee and Lobigs, which have identified residues in MVEV and JEV, two flaviviruses closely related to ALFV. In these studies, E protein residues E306 (JEV) and E390 (MVEV) were identified as being motifs for enhanced GAG binding. On cells, enhanced levels of GAG binding act to concentrate virions at the cell surface increasing virion avidity and enhancing the infection rate. This results in higher than normal initial replication rates in cell lines expressing relatively high levels of GAG, such as SW13 cells or BHK cells. Due to sulphate impurities in the agar overlay, viruses with a high affinity for GAG typically produce small plaques in plaque assay, and recent studies have indicated that the presence of GAG binding motifs may result in a reduced hemagglutination pH-range [34]. In vivo, however, enhanced affinity for GAG results in a reduction in peripheral replication and spread as virions are sequestered by the GAG-rich extracellular matrix and removed from the circulatory system by GAG-rich organs such as the liver and spleen [35]. To date flaviviruses with enhanced affinity for GAG have been produced by serial passage through BHK, and SW13 cells lines and through ticks. It is hypothesised that a GAG binding phenotype would be unable to arise in the wild as their attenuation in whole animal systems would interfere with the life cycle of the virus [34,35].

GAG binding in MVEV has been shown by Lee and Lobigs to be associated with residue 390 in domain three. In wild type virus this is a positively charged lysine residue; however, it is directly adjacent to a negatively charged aspartic acid residue (392), negating the net positive charge [36]. Passage through SW13 cells exerts a selective pressure toward GAG binding and results in exchange of aspartic acid at 392 for histine, glycine, serine or asparagines, resulting in a net increase in charge in the region [36]. Additionally, it was found through point mutagenesis that when residues with large sidechains were introduced at residue 392 GAG binding was again reduced, suggesting that protein conformation may play a role in virus attachment to GAG [36]. In JEV it was found that passage through SW13 cells introduced a positively charged residue at position 306 in domain three [35]. Both of these sites are physically adjacent to each other in the 3D structure of E, despite being 84 amino acid residues apart on the amino acid chain.

In this study we identified and confirmed that positively-charged residue (K327) plays a role in enhanced GAG binding in ALFV. Furthermore, residues E327, E273-277 and E154-156 were also confirmed to play a role in the natural attenuation of ALFV_3929_. These findings provide new insights for the rational design of attenuated flavivirus vaccines.

## 2. Materials and Methods

### 2.1. Cell Culture

Baby hamster kidney (BHK) cells, porcine stable-equine kidney (PS-EK) cells, Cos-7 cells and African green monkey kidney (Vero) cells were revived from frozen laboratory stocks and cultured in Dulbecco’s modified eagle medium (DMEM) (Gibco; Thermo Fischer Scientific, Australia ) supplemented with 2% fetal bovine serum (FBS) (Gibco). SW13 cells were cultured in DMEM supplemented with 10% FBS. C6/36 cells were cultured in RPMI media containing 10% FBS. All cultures were supplemented with the addition of 50 U/mL penicillin, 50 μg/mL streptomycin and 50µL/mL Glutamax (Gibco). All cells were passaged by dissociating the cell monolayer from the flask with trypsin/PBS (BHK and C6/36) or trypsin/EDTA (PS-EK and Vero) and vertebrate cells cultured at 37 °C with 5% CO_2_, while insect cells were cultured at 28 °C with 5% CO_2_.

### 2.2. Virus Culture and Titration Using TCID_50_

Stocks of ALFV and MVEV strains (see Table 1) were produced by infecting a sub-confluent monolayer of PS-EK cells with virus (multiplicity of infection [m.o.i.] 0.1) in DMEM containing 2% FBS. Culture supernatant was harvested after two to three days, when cytopathic effect (CPE) was evident in approximately 80% of cells and stored at −70 °C. ALFV virus strains K37414 and CY2269 were selected for testing due to their known passage history and geographically separated areas of isolation.

Virus stocks were titrated by serial 10-fold dilution in RPMI containing 2% FBS and used to infect C6/36 cells in 96 well plates (10 wells per dilution) in triplicate. Viral replication was determined by evidence of CPE 7 days post inoculation and confirmed by ELISA following fixation with 20% acetone, 0.02% BSA in PBS. TCID_50_ values were calculated using the Reed-Muench method [37].

**Table 1:** Virus isolates used in this study.

Strain	Year of Isolation	Place of Isolation	Source of Isolation	Passage History	Reference
ALFV MRM3929 ^1^	1966	Kowanyama, QLD ^2^	*Centropus phasianius* ^3^	Unknown	[38]
ALFV K37414	1999	Wyndham, WA ^2^	*Culex pullus* ^4^	2 C6/36, 3 PS-EK	
ALFV CY2269	1999	Pompuraaw, QLD ^2^	*Culex annulirostris* ^4^	1 C6/36, 3 PS-EK	
MVEV 1–51	1951	Mooroopna, Vic ^2^	Fatal human case	Unknown	[39]

^1^ Prototype strain of virus; ^2^ QLD, Queensland; WA, Western Australia; Vic, Victoria; ^3^ Pheasant coucal, a cuckoo species endemic in Northern Australia; ^4^ Species of mosquito.

### 2.3. Serial Passage of Virus

SW13 cell monolayers in 6-well plates were infected with virus at a m.o.i. of 0.1, and supernatants were collected and supplemented with FBS (10% final concentration) when significant cytopathic effect was observed. Five subsequent passages were performed for each series at the same m.o.i., harvesting culture medium each time upon observing significant cytopathic effect.

### 2.4. Construction of pMVEV/ALFVstr

The infectious clone, pMVEV/ALFVstr was constructed by exchanging the prM and E genes of the pMVEV 1−51 plasmid reported elsewhere [40] with the prM and E genes of ALFV, obtained by PCR from viral RNA as previously described (15). For the construction of mutant viruses, mutations in the glycosylation (E154–156), hinge region (273–277) and putative GAG-binding site (E327) were introduced by overlap extension PCR mutagenesis using Phusion polymerase (Finnzymes; Thermo Fischer Scientific, Australia) as previously described [15,41]. The nucleotide sequences of primers used for mutagenesis are shown in Table S1. Plasmids containing the desired mutations were confirmed by sequence analysis (Australian Genome Research Facility, The University of Queensland, Australia). Primers used in plasmid construction and for sequence confirmation were designed using Invitrogen’s Vector NTi software and ordered from Intergrated DNA Technologies (Coralville, IA, USA) (Table S1).

### 2.5. RNA Electroporation

cDNA was linearized by overnight digestion with Bsu36I and purified by phenol/chloroform extraction. RNA was in vitro transcribed from 1 μg of linearized cDNA using 10 mM DTT, 5 mM of rATP, rUTP and rCTP, 1 mM rGTP (Promega, Sydney, Australia), 4 mM cap analogue (NEB, Ipswich, MA, USA), 20 U Rnasin (Promega) and 100 U T7 RNA polymerase (Promega) as previously described [42]. The reaction mix was incubated at 37 °C for 2 h then treated with RNase-free DNase. To confirm transcription, 1µL of product was run on a 0.5% RNase-free agarose gel. Trypsinised BHK cells were washed three times in ice-cold DEPC-PBS and resuspended in DEPC-PBS to a cell density of 5 × 10^6^ cells/mL. The RNA and 400 μL cells were added to a 0.2 cm Genepulser cuvette (BioRad, Gladesville NSW, Australia) and pulsed twice at 1.5 kV, 25 μF and ∞ Ω in a Genepulser II electroporator (BioRad). Cells were resuspended in 10 mL DMEM containing 10% FCS, added to a 75 cm^2^ flask and grown at 37 °C with 5% CO_2_. The cells were monitored for signs of cytopathic effect (CPE), and supernatants were harvested 3 and 6 days post-electroporation.

### 2.6. DNA/RNA Sequencing

PCR products were sequenced using BigDye technology and processed by Sanger sequencing by the Australian Genome Research Facility (AGRF). The resulting chromatograms were analysed using Contig Express (Vector NTI, Informax, Gaithersburg, MD, USA).

Viral RNA was extracted from samples using the Macherey Nagel Nucleospin RNA Virus isolation kit, following the manufacturer’s protocol. First strand cDNA was transcribed using Superscript III (Invitrogen, Carlsbad, CA, USA) according to the manufactures instructions and amplified using Phusion (Finnzymes, Thermo Fischer Scientific, Queensland, Australia) and sequenced by AGRF. The resulting chromatograms were analysed using Contig Express (Vector NTI, Informax, Gaithersburg, MD, USA).

### 2.7. Plaque Assay/Purification

Cells were cultured in 6 well plates, until 85–90% confluent. Subsequently they were infected with a series of ten-fold dilutions and incubated for one hour. The inoculums were then removed and the monolayer rinsed with PBS. Cells were overlaid with 0.75% low melting point agarose in DMEM (2% FBS) and incubated at 37 °C. At three days post infection, monolayers were fixed with 10% formaldehyde and stained with 0.2% crystal violet. In the case of viruses that were plaque purified, at two days post infection agar plugs were overlaid with 0.75% low melting point agarose in DMEM (2% FBS) with the addition of 0.02% Neutral Red. Plaques were harvested using 200 µL tips to aspirate the agar over the plaque. Plaque purified samples were passaged once over C6/36 cells to produce a working stock.

### 2.8. Viral Growth Kinetics

Cells were cultured in the wells of a 24 well plate, allowing triplicate wells per time point per virus. When the cells were sub-confluent they were infected with virus at an multiplicity of infection (m.o.i.) of 0.1, diluted in a 500 μL volume of DMEM containing 2% *w/v* FBS for one hour at 37 °C. Post incubation virus inoculate was removed and the cells were washed with PBS before addition of 1 mL DMEM containing 2% *w/v* FBS prior to incubation at 37 °C for 72 h. Two 500 μL samples were removed from the assigned wells every 12 h p.i. and stored at −70 °C. The virus titre in each sample was determined by TCID_50_ on C6/36 cells.

### 2.9. Heparin Inhibition Assay

Cells were cultured on 96 well plates until ~70% confluence had been achieved; cell monolayers were then preincubated with heparin (Sigma) at concentrations of either 0, 50 or 200 µg/mL diluted in Hank’s buffered saline solution (HBSS) supplemented with 0.2% bovine serum albumen (BSA) *w/v* for 30 min at 4 °C prior to the addition of virus.

Viral samples were preincubated in serial ten-fold virus dilutions (10^−1^ to 10^−8^) with heparin (0, 50, 200 µg/mL) diluted in HBSS supplemented with 0.2% BSA *w/v* for 30 min at 4 °C. Prior to incubation with the virus samples, preincubated supernatant was removed from each well and replaced with 50 µL of viral diluents. Ten replicates per dilution were incubated at 4 °C for 30 min. Cell monolayers were then rinsed in ice cold PBS and 100 µL of DMEM supplemented with 2% fetal bovine serum (FBS) *w/v* added to each well. Plates were incubated for 7 days and then examined for cytopathic effect, fixed and analysed via fixed cell ELISA and scored according to the method proscribed by Reed and Muench [37]. Graphs were plotted using Microsoft Excel with standard error of the replicate values for each sample. Inhibition was then calculated as a percentage difference between no heparin and heparin at 50 and 100 µg/mL.

### 2.10. ELISA

Fixed cells were blocked with blocking buffer (0.05 M Tris/HCl (pH 8.0), 1 mM EDTA, 0.15 M NaCl, 0.05% (*v*/*v*) Tween-20, 0.2% *w/v* casein) for 1 h at 28 °C before addition of the primary antibody. For antigenic analyses, mAbs were diluted 1/10, then serially diluted tenfold in blocking buffer. Mouse sera were diluted 1/10 followed by serial 2-fold dilutions, also in blocking buffer. The antibody dilutions were added to the wells and incubated at 28 °C for 1 h. The plates were washed 4 times with PBS containing 0.05% Tween-20 (PBS-T) and tapped dry before addition of goat anti-mouse HRP-conjugate (Dako; Agilent, Santa Clara CA, USA), diluted 1/2000 in blocking buffer. This was again incubated for 1 h at 28 °C, before washing 6 times with PBS-T. Three mM hydrogen peroxide and 1 mM ABTS (2,21-azino-bis (3-ethylbenzthiazoline-6-sulfonic acid)) in 0.1 M citrate/0.2 M Na_2_PO_4_ buffer pH 4.2 was added to each well. After being allowed to develop in the dark for 1 hr, the absorbance was read on a Multiscan EX plate reader (Labsystems) at 405 nm. Reactions were considered positive when the absorbance was at least 0.25 OD units and at least twice the absorbance of the corresponding dilution on an uninfected control plate.

### 2.11. Mouse Virulence

All animal procedures had received prior approval from The University of Queensland Animal Ethics Committee and where necessary were performed under ketamine:xylazil anaesthesia. Three-week old Swiss outbred mice (Animal Resources Centre, Murdoch, Western Australia, Australia) were infected intraperitoneally (i.p.) with a range of doses of wild type, mutant or chimeric viruses. Mice were kept on clean bedding and given food and water ad libitum. Infected animals were monitored daily for the onset of clinical signs and culled when the first signs of encephalitis (hunching, lethargy, eye closure, or hind leg flaccid paralysis) were apparent. Surviving mice were bled by cardiac heart puncture at the end of the experiment (Day 21) and the sera were tested for evidence of seroconversion as previously described [43]. The significance of clinical differences between groups was calculated by Kaplan-Meier analysis where noted (GraphPad Prism Version 5.0, GraphPad Software Inc., San Diego, CA, USA).

## 3. Results

### 3.1. Low Passage ALFV Isolates Exhibit a Mixed Plaque Morphology

ALFV_3929_ is the prototype laboratory strain; however, due to its largely unknown passage history, it may not represent the wild type ALFV phenotype. Therefore, two low-passage ALFV isolates (K37414 and CY2269) with a defined passage history were included in the study. To ensure that the populations were clonal, each virus was first characterised via plaque assay on PS-EK cells. This revealed that while both low-passage ALFV strains had predominantly large plaques (~3 mm), a mixed population was observed for each with ~10% of the plaques relatively small (~1 mm) (Table 2). By comparison ALFV_3929_ (~1 mm) and MVE/AFVstr (~1.5 mm) displayed uniform small plaques while MVEV produced only large plaques (~3 mm). Small and large plaques were purified for each of the low passage ALFV strains to produce large plaque (K37414L and CY2269L) and small plaque (K37414S and CY2269S) variants of each isolate.

### 3.2. Small-Plaque ALFV Variants Exhibit Enhanced Glycosaminoglycan (GAG) Binding

**Figure 1:**
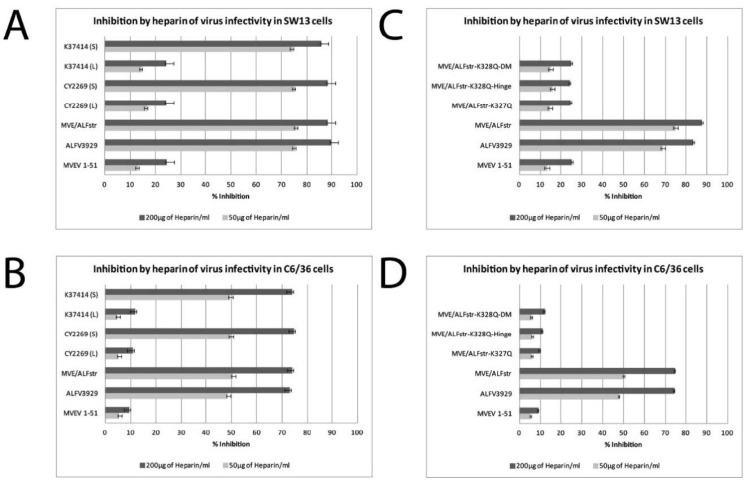
Heparin inhibition by of ALFV_3929_ and other small plaque ALFV derivatives. Viruses were incubated with heparin (50 µg/mL or 200 µg/mL) for 15 min prior to their addition to SW13 (**A**,**C**) or C6/36 (**B**,**D**) cell monolayers. Error bars indicate the standard deviation values calculated for each triplicate. DM stands for the double mutant which contains the MVEV sequence in both the hinge and glycosylation sites. Percentage inhibition of titre caused by heparin was calculated as follows: 100 − (TCID_50_ in the presence of heparin/TCID_50_ of mock treated control) × 100%. MVE/ALFVstr-K327Q-DM = MVE/ALFstr-CHO+/H**^MVEV^**/K327Q, MVE/ALFstr-K327Q-Hinge = MVE/ALFstr-H**^MVEV^**/K327Q.

The small plaque phenotype observed for ALFV_3929_, ALFV_CY2269_S ALFV_K37414_S and MVE/ALFstr, was assessed for GAG binding alongside the large-plaque viruses MVEV, ALFV_CY2269_L and ALFV_K37414_L using a modified heparin sensitivity assay. This assay quantifies GAG binding of viruses, with a higher affinity to GAG displayed by reduced infectivity in the assay and a reduction in the plaque count. It was found that the incubation of 50 µg of heparin with ALFV_3929_, MVE/ALFVstr and small plaque ALFV clones resulted in a 79–84% reduction on SW13 cells and 45–50% reduction on C6/36 cells (Figure 1A,B). In contrast when MVEV, and large plaque ALFV clones were incubated with 50 µg of heparin on these cell types a reduction in titre of only 12% on SW13 cells and 5% on C6/36 cells (Figure 1A,B) was observed. When incubated with 200 µg of heparin during inoculation, ALFV_3929,_ MVE/ALFVstr and small plaque ALFV clones resulted in a reduction in titre of >80% on PS-EK and SW13 cells and >70% on C6/36 cells, while MVEV and large plaque ALFV clones resulted in a reduction of <30% on PS-EK cells and SW13 cells under the same reaction conditions (Figure 1A,B). In summary these results indicate a significantly increased affinity for GAG for ALFV_3929_, MVE/ALFVstr and the small-plaques ALFV variants when compared with MVEV and the large plaques viruses (<0.0001 as determined by two-tailed, unpaired, *t*-tests comparing relevant pairs) (Table S2).

### 3.3. A Novel GAG-Binding Motif Maps to Residue 327 in the ALFV E Protein

Sequence analysis of EDIII domains of ALFV_3929_ and MVEV revealed that there was no difference in sequence at the RGD motif, nor in the flanking regions of the amino acid chain. Thus, it was concluded that the RGD motif was not involved in ALFV GAG binding, in contrast to GAG-binding variants of MVEV (14) (Table 3). However, sequence analysis of the plaque purified ALFV isolates showed only one difference in the E protein at residue 327. At this residue in ALFV_3929_ and the small plaque variants of CY2269 and K37414 there is a lysine residue, an amino acid with a highly positively charged side chain. In the large plaque variants and MVEV the residue in this location (glutamine) has a neutral charge (see Table 3).

**Figure 2:**
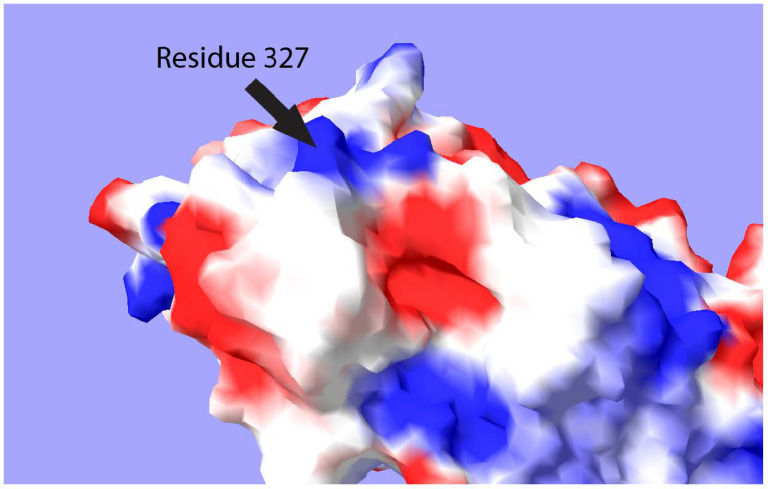
Computer generated map of the surface of E protein Domain III. Surface map was generated by Swiss-Pdb-Viewer Ver.4.0.4. Electrostatic potential was calculated using the Poisson-Boltzmann method. Blue indicates a positive charge, while red indicates a negative charge.

Using the program Swiss-Pdb-Viewer, the surface of the ALFV envelope protein was modelled and the electrostatic potential of the surface residues mapped to it using the Poisson-Boltzmann method. It was found that ALFV E protein residue 327 is surface expressed in the resting conformation of the protein and exhibits a strong positive charge, typical of a GAG-binding motif (Figure 2).

### 3.4. Construction and Characterisation MVE/ALFstr Mutant Constructs

To substitute ALFV E reside 327 (K) for that of MVEV E residue 327 (Q), a point mutation was carried out using overlap extension mutagenesis. In brief, the nucleotide sequence AAG was changed to CAG at positions 1975 to 1977, to convert the Lys residue to a Gln residue. This mutation was introduced into the MVE/ALFstr chimera (15), and into previously described variants of this chimera containing the hinge (E273–277) and/or the glycosylation motif (E154–156) derived from MVEV (15). After sequence confirmation of each mutant construct, their plaque phenotype was assessed on PS-EK cells. As predicted, mutant constructs with lysine exchanged for glutamine at residue 327 produced large plaques with a 3 mm diameter, similar to that produced by MVEV. As with previous experiments ALFV_3929_ and MVE/ALFVstr both produced small plaques (1–1.5 mm).

Heparin sensitivity assays were also performed for the residue 327 MVE/ALFVstr mutant constructs and their parental viruses (MVEV, MVE/ALFstr and ALFV_3929_) as described above. Heparin was shown to inhibit infectivity of ALFV_3929_ and the parental chimera by a maximum of ~84–90% on SW13 cells. In contrast the residue 327 mutant constructs were only inhibited by ~23–25% on SW13 cell lines, comparable with MVEV (Figure 1C,D).

**Figure 3:**
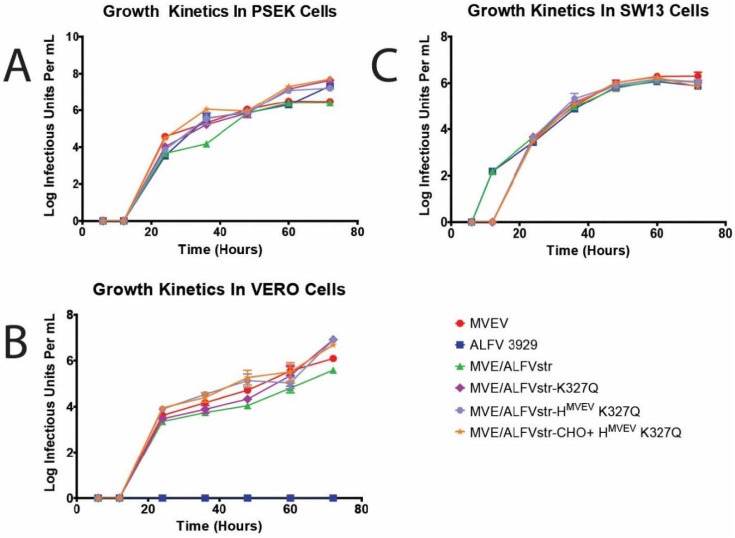
Growth kinetics of glycosaminoglycans GAG mutants. Growth kinetics of Murray Valley encephalitis virus (MVEV), ALFV3929 MVE/ALFVstr and associated mutants on PS-EK (**A**), Vero (**B**) and SW-13 cells (**C**). Cells infected at an m.o.i of 0.1, viral titre determined by TCID_50_ on C6/36 cells.

Replication kinetics of the residue 327 mutant constructs were assessed in PS-EK, Vero and SW13 cells, and compared with their parental strains (MVEV, ALFV_3929_, and MVE/ALFstr). All mutants replicated with similar efficiency to their parental strains (Figure 3). However, in SW13 cells, MVEV and the mutant constructs exhibited a delay in replication of approximately six hours when compared with ALFV_3929_ and MVE/ALFVstr, which is characteristic for a virus that lacks GAG binding.

### 3.5. Survival Assay (In Vivo)

**Figure 4:**
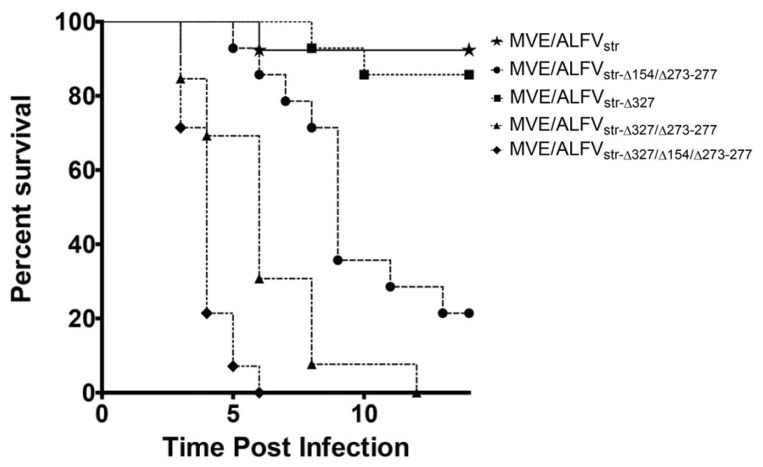
Kaplan-Meier survival curves of Swiss mice inoculated i.p. with 10^5^ infectious units with MVEV/ALFVstr and associated mutants. (*n* = 10) *p*-Value is 0.0195. Curves produced using GraphPad Prism Ver.5.0.

To assess the effect of GAG binding, glycosylation of ALFV E protein and substitution of the distal hinge region motif on neuro-invasion, three weeks old Swiss white mice were inoculated i.p. with parental and mutant viruses at 10^5^ IU. Mice infected with the GAG binding knock-out mutants containing the MVEV–like distal hinge region substitution (mutants containing Δ327) exhibited a significantly higher mortality rate than mice infected with the parental virus (*p* < 0.05) (Figure 4). Introduction of a glycosylation motif in E in addition to GAG knockout and MVEV-like distal hinge region MVE/ALFstr-CHO+/H^MVEV^/K327Q ) also resulted in a significantly higher mortality rate than that caused by the parental virus (*p* < 0.05), in addition to being significantly faster in causing mortality than GAG knock-out combined with MVEV-like distal hinge region MVE/ALFstr-CHO+/H^MVEV^/K327Q. Interestingly, both mutant constructs caused significantly higher and faster mortality than a construct with glycosylation of E and substitution of MVEV-like hinge region. In contrast, removal of the GAG-binding motif alone did not significantly alter mortality when compared to the parental virus (Figure 4). These results suggest that, in isolation, GAG binding has no discernable effect on ALFV neuro-invasion. However, removal of GAG binding in conjunction with substitution of the hinge region with that of the corresponding sequence of MVEV significantly increases neuro-invasion. Additionally, the combination of glycosylation of E and MVEV-like distal hinge region in a GAG knock-out construct appears to speed up neuro-invasion and the onset of encephalitis. However, it should be noted here that CNS titers were not determined for mice that succumbed to infection in these experiments. While we have previously shown that wild type ALFV replicates efficiency in the brain after IC inoculation and presume that attenuation is due predominantly to motifs that reduce neuro-invasion, we cannot exclude the possibility that the differences in mortality rates and time to death between the mutants tested here are not partially due to differences in neurovirulence (the ability of the virus to replicate in neural tissue).

An unusual clinical sign of encephalitis was also observed in this study. In most cases mice developing encephalitis due to a neuro-invasive flavivirus species display a period of impaired grooming/ruffled fur before more severe neurological signs manifest (trembling, spasms and seizures). In this study mice infected with mutants derived from MVE/ALFVstr displayed incessant grooming behaviour before the onset of more severe clinical signs. In this state mice groomed continuously, ignoring all but the most invasive interference (i.e., picking the subject up by its tail). As soon as the interference was removed, the mouse returned immediately to its grooming behaviour.

A few mice with this clinical presentation were subjected to histopathological examination and were found to have moderate to severe meningo-encephalitis. One animal surviving until termination of experiment (day 20 p.i.) had severe granulomatous meningo-encephalitis with mineralization of the cerebral cortex (Figure S1).

## 4. Discussion

In this study we identified a novel flavivirus attenuation marker in the EDIII of ALFV. The substitution of glutamine with a large positively charged residue (lysine) at E327, substantially enhanced GAG binding and reduced plaque size in ALFV. This substitution also reduced neuro-invasiveness of infectious clones containing the ALFV prM-E genes in an MVEV genome backbone.

This extends our understanding of the mechanisms of attenuation in ALFV and the JEV serogroup of flaviviruses. Previously, we showed that ALFV is significantly less neuro-invasive than MVEV but is only slightly less neurovirulent (1). This indicates that ALFV is able to replicate efficiently in the brain after IC inoculation but is unable to invade the central nervous system after IP inoculation. Subsequently, we established that the distal hinge region of ALFV (E273–277), and the lack of glycosylation at E154 were major attenuation motifs but were insufficient to explain the complete lack of neuro-invasion by ALFV in the murine model (15). Initially we excluded an additional role for GAG binding due to the lack of a substitution at the RGD motif at residue 390, a major GAG binding motif in the closely related MVEV. However, other data suggested ALFV may possess cryptic GAG binding sites.

Our inability to detect infectious virus in the serum or peripheral organs of mice after inoculation with ALFV_MRM3929_ (1) suggested an inability of the virus to spread from the site of inoculation, which may contribute to a lack of neuro-invasion, as seen in other studies [35,44,45]. Previously, Lee and Lobigs (2002) have shown that substitutions at residue 390 in the conserved RGD motif in the E protein of MVEV promotes high-affinity binding to glycosaminoglycans (GAG) on cellular surfaces resulting in the rapid removal of virus from the blood by GAG rich tissues and a loss of neuro-invasiveness in mice. While the RGD motif is conserved in ALFV, the presence of a positively charged residue on the surface of domain III (K327) that promotes GAG-binding provides an explanation for the absence of virus in the blood and lack of neuro-invasion for mice infected with ALFV or viruses containing the ALFV E protein.

A high GAG affinity results in a reduced plaque size due to sulphate impurities in the agar overlays, which sequesters GAG binding viruses [46,47]. Therefore, our demonstration that the highly passaged prototype ALFV_3929_, and its chimeric derivative (MVE/ALFstr), show uniformly small plaques compared to low passage ALFV isolates, that predominantly exhibit large plaques, suggests that the GAG binding phenotype maybe a result of laboratory passage of ALFV_3929_. Serial passage has long been an established means of attenuating viruses and is still used frequently today in the production of live attenuated vaccine strains [48,49]. Due to the replicative advantages of binding to a secondary receptor to enhance virion affinity to target cells, mutations enhancing GAG binding allow variants to rapidly out-compete the original virus sub-species. We have shown that passage through either PS-EK cells or SW13 cells has the ability to exert selective pressure leading to the rise of ALFV variants with high affinity to GAG [50]. While SW13 cells have long been associated with this behaviour, this is the first time that PS-EK cells have been shown to influence the GAG affinity of flaviviruses.

While mutations due to cell passage in a laboratory setting rather than under natural transmission conditions are likely to provide no advantage to the life cycle of the virus, it is worth considering that serial passage through ticks also produced a high affinity GAG motif in TBEV in a study by Romanova et al. [51]. It should also be noted that very little is known about the replication of flaviviruses in the insect model, and it may be that high affinity to GAG does not affect the virus in the same manner as in vertebrate systems. The authors suggest that this might have particular significance to insect-specific flaviviruses as they do not require passage through a vertebrate host to complete their life cycle [52,53,54].

Residue 327 of the envelope protein has hitherto never been associated with GAG binding in flaviviruses; however, it is physically located on the surface of the E protein in a proximal location to reside 306 and residue 390 which have both been associated with GAG binding in previous studies [36]. Our results indicate that by replacing the positively charged lysine residue which occupies this motif in ALFV3929 with that of glutamine, which is found at this location in MVEV, GAG binding is significantly reduced, resulting in a larger plaque size and a delay in replication in SW13 cells. However, replication kinetics in the other tested cell lines remains identical to the parental viruses. While MVEV has a GAG binding motif at residue 390 in the RGD motif, its conformation places it close to the virion envelope in resting conformation. The positively charged residue responsible for GAG binding is present in wild type MVEV, and substitution of adjacent residues with smaller residues is required to make the motif surface accessible. We propose that this residue is unavailable in ALFV, as that the ALFV E protein has a number of residues in the DI-DIII linker region linking domains I and III which may alter E protein conformation, making it inaccessible due to is closer proximity to the envelope surface.

Prior studies have associated high GAG affinity in the E protein of flaviviruses with significant attenuation in vivo, probably due to released virions being sequestered by the GAG rich extracellular matrix and filtered from circulation by GAG rich organs such as the liver and spleen. However, our prior studies have shown that low passage field isolates of ALFV, that were shown to lack GAG binding in the present study, were also highly attenuated in mice (1). This indicates that GAG affinity alone does not account for ALFV attenuation in the murine model and suggests that ALFV attenuation is multi-factorial. Indeed, we found in this study that removal of GAG affinity from ALFV E protein of MVE/ALFstr did not significantly alter attenuation of neuro-invasion. However, when this substitution was made in conjunction with exchanging the distal hinge region in ALFV (E273–277) with that of MVEV, and creation of an N-linked glycosylation site at E154 it produced a mortality curve very similar to wild type MVEV in the murine model (1).

One lesson that can be taken from this study is that researchers must be careful when working with laboratory passaged strains. In this study we found that ALFV_3929_ had adapted to cell culture due to repeated passage since its isolation in 1966 and, as with many old laboratory strains, its passage history was unknown. Forensic analysis of early published data on ALF_3929_ suggests that it was initially passaged through mouse brains and PS cells which may have exerted selective pressure on the virus. Early data regarding haemagglutination and plaque size suggests that ALFV did not display an enhanced affinity GAG [55], although the lack of actual GAG inhibition studies at that time does not exclude that phenotype in the original isolate. However, it is unlikely that ALFV displayed GAG binding in the wild as this would significantly hamper its ability to replicate sufficiently in its natural avian hosts to sustain transmission to mosquitoes.

In conclusion, this study adds to our understanding of attenuation markers for ALFV and flaviviruses in general. Identifying novel motifs leading to attenuation could be incorporated into next-generation flavivirus vectors that provide a useful vehicle for safer vaccine candidates.

## Figures and Tables

**Table 2:** Plaque morphology of Alfuy (ALFV) strains and clones.

Virus Isolates & Clones	Plaque Phenotype Status	Plaque Size
ALFV_3929_	small	1 mm
ALFV_CY2269_	mixed	1/3 mm
ALFV_CY2269_ S	small	1 mm
ALFV_CY2269_ L	large	3 mm
ALFV_K37414_	mixed	1/3 mm
ALFV_K37414_ S	small	1 mm
ALFV_K37414_ L	large	3 mm
MVEV	large	3 mm
MVE/AFVstr ^1^	small	1.5 mm

^1^ Structural (prM-E) genes derived from ALFV_3929_.

**Table 3:** Sequence analysis of the E gene in plaque variants of ALFV identifies a substitution at E327.

Virus Isolates	Glycosylation Motif E154–156	Hinge Region E273–277	K327Q Substitution Site E325–330
ALFV_3929_	DYS	QMDS-T	ELKYL
ALFV CY2269 (L)	DYS	QMDS-T	ELQYT
ALFV CY2269 (S)	DYS	QMDS-T	ELKYL
ALFV K37414 (L)	DYS	QMDS-T	ELQYT
ALFV K37414 (S)	DYS	QMDS-T	ELKYL
MVEV_1–51_	NYS	EFSSST	ELQYT
MVE/AFVstr-K327Q	DYS	QMDS-T	ELQYT
MVE/AFVstr-H^MVEV^/K327Q	DYS	EFSSST	ELQYT
MVE/AFVstr-CHO^+^/H^MVEV^/K327Q	NYS	EFSSST	ELQYT

L = large plaque morphology, S = small plaque morphology.

## Data Availability

Not applicable.

## Supplementary Materials

The following are available online at https://www.mdpi.com/1999-4915/13/2/147/s1, Table S1: Primers used for mutagenesis and sequencing.
