# Supplementary material for: Novel Flavivirus Attenuation Markers Identified in the Envelope Protein of Alfuy Virus
Source: Viruses. 2021 Jan 20;13(2):147. doi: 10.3390/v13020147 (PMC7909262; doi:10.3390/v13020147)
Supplement: Supplementary file 1 [file viruses-13-00147-s001.zip › viruses-1058968-for conversion/Supplementary Material for Resubmission/Figure S1.pdf]

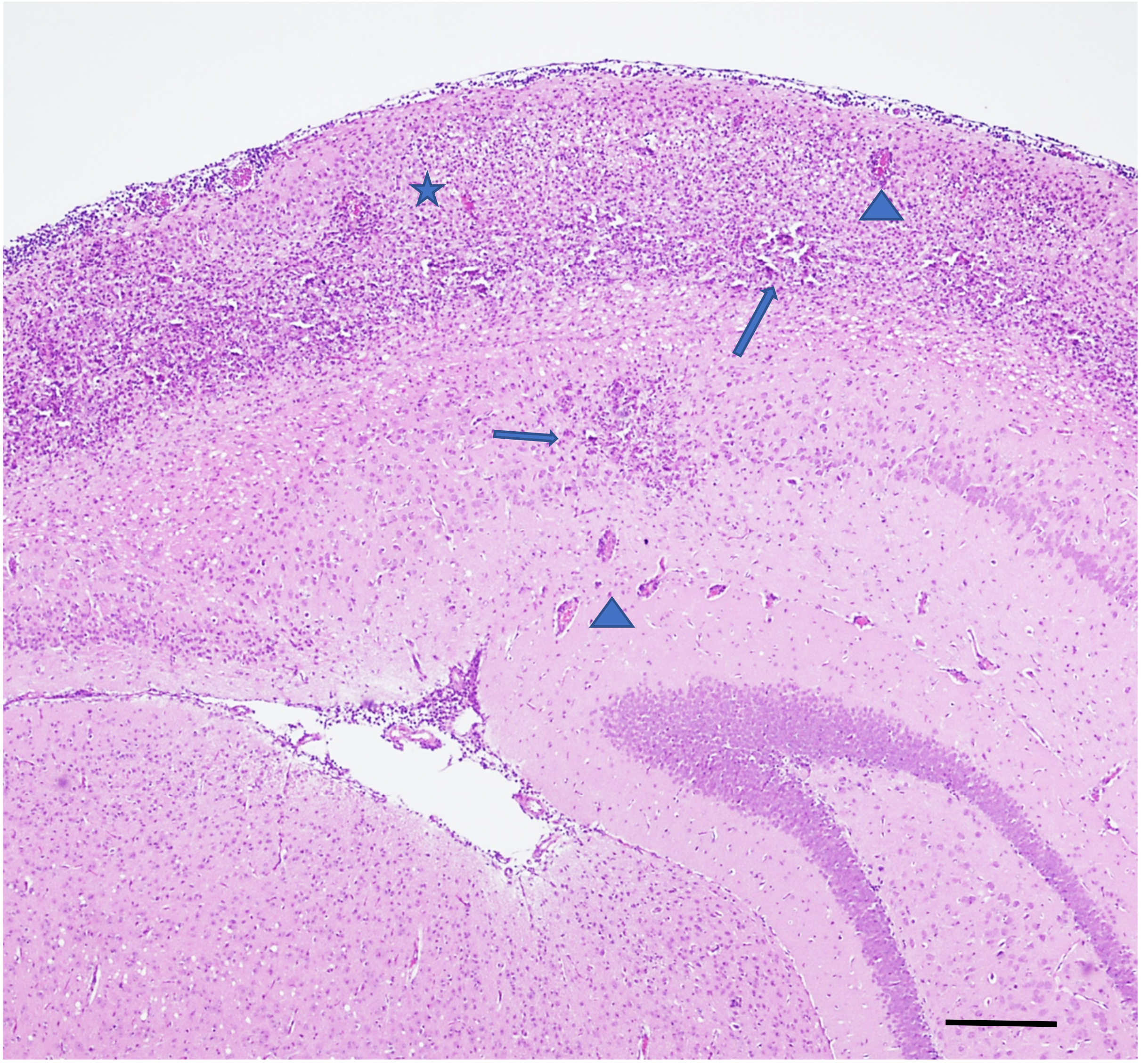

Supplementary Figure 1. Cerebral cortex of mouse infected with GAG-mutant. The outer neuronal layers are diffusely infiltrated with macrophages and lymphocytes (star), there is almost complete loss of neurons, vascular cuffing (arrow heads) and multifocal mineralization of the inflamed neuropil (arrows). H&E stained paraffin-embedded tissue section. Scale bar: 200 µ
