# Supplementary material for: Novel Flavivirus Attenuation Markers Identified in the Envelope Protein of Alfuy Virus
Source: Viruses. 2021 Jan 20;13(2):147. doi: 10.3390/v13020147 (PMC7909262; doi:10.3390/v13020147)
Supplement: Supplementary file 1 [file viruses-13-00147-s001.zip › viruses-1058968-for conversion/Supplementary Material for Resubmission/Table S1.pdf]

Supplementary Material

Table S1. Primers used for mutagenesis and sequencing

Oligo Name	Type	Sequence (5' - 3')	Gene
**MVE832s (Ff)**	Forward	GGATTCCACCAAGGCCACTCG	C
**MVE4450 (Fr)**	Reverse	CGCTGGTACCTGTTATTGCTGCC	NS1
**ALF1450ΔGlyΔXmn1s**	Mutagenesis	CCATGGA**A**ACTA**T**TCCACCCAAGTAGG	E
**ALF1476ΔGlyΔXmn1**	Mutagenesis	CCTACTTGGGTGGA**A**TAGT**T**TCCATGG	E
**ALF1801ΔHinΔXho1s**	Mutagenesis	AGTGCAG**TTCTCGAGC**AGCACATTGAAG	E
**ALF1835ΔHinΔXho1**	Mutagenesis	CTTCAATGTGCT**GCTCGAGA**ACTGCACT	E
**MVE908s**	Forward	GCTGTAGCTCTAGGCTGGATGC	C
**MVE4380**	Reverse	GGCTTTTCCCGAGATAACATAGG	NS1
**pALF3548F**	Sequencing	GTCGCAGGAGGGAGCACTTCA	E
**ALFE17125**	Sequencing	GCCACTAAGCAATCCGTGGTCGC	E
**ALFE1549**	Sequencing	TGGTTCGCATTCCACTGTCAC	E
**ALF327-GAG-F**	Mutagenesis	TAGTGTTAGAACTCAAGTATTTGGGAGC	E
**ALF327-GAG-R**	Mutagenesis	TCCCAAATACTTGAGTTCTAACACTACC	E
**pMVE/ALFE-F**	Mutagenesis	GATTAAGTTGGGTAACGCCAGG	E
**pMVE/ALFE-R**	Mutagenesis	GAAGCACCTTCTAGGAAATCACG	E

Nucleotides in Bold indicate MVEV sequence
